# Bacterial Communities Associated with Whole Eggs and Gut Tissues of *Heortia vitessoides* Moore (Lepidoptera: Crambidae) Across Developmental Stages and Sexes

**DOI:** 10.3390/insects17070668

**Published:** 2026-06-26

**Authors:** Pengfei Zhao, Mingshan Chang, Xuejian Jiang, Ji Luo, Qingdong Xiang, Zhixing Lu, Youqing Chen

**Affiliations:** 1 Institute of Highland Forest Science, Chinese Academy of Forestry, Kunming 650224, China; 2 College of Forestry and Grassland, Nanjing Forestry University, Nanjing 210037, China; 3 Guangxi Laboratory of Forestry, Nanning 530002, China; 4 Guangxi Key Laboratory of Special Non-Wood Forests Cultivation and Utilization, Nanning 530002, China; 5 Guangxi Key Laboratory of Superior Timber Trees Resource Cultivation, Nanning 530002, China; 6 Guangxi Forestry Research Institute, Nanning 530002, China

**Keywords:** insect microbiota, *Heortia vitessoides*, metamorphosis, moth, *Aquilaria* trees

## Abstract

Bacterial communities may affect insect nutrition and development, but their variation across complete metamorphosis remains poorly understood in many forest lepidopteran pests. *Heortia vitessoides* is an important defoliator of *Aquilaria* trees, which produce agarwood. In this study, we used 16S rRNA gene sequencing to characterize bacteria associated with whole eggs and gut tissues from larvae, pupae, and adults of *H. vitessoides*. Whole egg and larval gut samples generally showed higher bacterial richness and diversity than pupal and adult gut samples. Bacterial community composition differed among the six groups examined, including whole eggs, larval guts, male pupal guts, female pupal guts, male adult guts, and female adult guts; a separate analysis of gut samples also indicated differences among larval, pupal, and adult gut communities. Proteobacteria, especially *Acinetobacter*, were abundant in whole egg and larval gut samples, whereas adult gut samples were dominated by Firmicutes, particularly *Enterococcus*. Sex-related differences were mainly observed in pupae, with *Methylobacterium* and *Pseudomonas* showing higher relative abundances in female pupae. PICRUSt2 predicted functional categories mainly related to nutrient metabolism and membrane transport. In a preliminary egg surface sterilization assay, larvae from treated eggs showed longer larval and total developmental durations than controls. Overall, these results provide baseline information on bacteria associated with whole eggs and gut tissues of *H. vitessoides* and identify candidate taxa for future functional studies.

## Simple Summary

Microorganisms are closely associated with insects and may affect their development, nutrition, and adaptation to host plants. *Heortia vitessoides* is a serious defoliating pest of *Aquilaria* trees, but little is known about the bacteria associated with this insect during development. In this study, we examined bacteria associated with whole eggs and gut tissues from larvae, pupae, and adults. Whole egg and larval gut samples generally contained richer bacterial communities than the pupal and adult gut samples. The dominant bacterial groups also differed among samples: Proteobacteria were more common in whole eggs and larval guts, whereas Firmicutes dominated adult guts. Differences between sexes were more evident in pupae than in adults. In a preliminary egg surface sterilization assay, larvae from treated eggs showed longer larval and total developmental durations than untreated controls. These findings provide basic information on bacteria associated with *H. vitessoides* and offer a starting point for future studies on insect–microbe interactions in this pest.

## 1. Introduction

*Heortia vitessoides* Moore (Lepidoptera: Crambidae) is an economically significant defoliating pest reported from Southeast Asia, Australia, and China [1,2,3]. The species is oligophagous and shows a strong host preference for trees of the genus *Aquilaria* (Thymelaeaceae) [4,5]. *Aquilaria* species produce agarwood, a resinous, fragrant, and highly valued heartwood widely used in traditional medicine, incense, and high-end perfumery [6]. This precious forest product has long attracted considerable research attention. Larvae of *H. vitessoides* feed voraciously on *Aquilaria* leaves and can rapidly defoliate seedlings and young trees [7]. Such herbivory not only causes severe economic losses to the agarwood industry, but also threatens the conservation of endangered *Aquilaria* species [8,9]. Because insect-associated bacteria are often linked to host nutrition, feeding performance, and adaptation to plant diets [10], bacterial communities offer a useful entry point for understanding the biology of this forest pest.

The gut microbiota has been linked to the fitness of lepidopteran hosts and may influence development, nutrition, immunity, and adaptation to the environment [11,12]. Lepidopteran larvae mainly feed on plant tissues, which are often nutritionally unbalanced and contain structural compounds and defensive secondary metabolites. Gut bacteria may contribute to the use of plant-derived substrates by providing digestive enzymes, producing metabolites, modulating host immunity, or participating in the transformation of plant secondary compounds and xenobiotics [13,14]. These bacterial communities are shaped by diet, temperature, environmental microbes, gut morphology, gut pH, and antimicrobial peptides [15]. However, most studies on lepidopteran-associated bacteria have focused on feeding larvae or adults. Much less is known about how bacterial communities differ among eggs, larvae, pupae, and adults of insects with complete metamorphosis [16]. This limits our understanding of how bacteria are maintained, removed, or reassembled during the transitions from egg to larva, pupa, and adult.

Complete metamorphosis is accompanied by major changes in gut structure, feeding activity, and host physiology, all of which can influence the persistence and reassembly of insect-associated bacterial communities [17]. Bacteria detected in eggs may originate from the mother, the egg surface, host plants, the rearing substrate, or the surrounding environment, and some of these bacteria may contribute to early larval colonization [18,19,20]. The transition from a feeding larva to a pupa is likely to be an important developmental bottleneck because the gut is extensively remodeled and transient bacteria introduced with food may be reduced or removed [21]. After adult emergence, the bacterial community may change again as feeding behavior, gut physiology, dispersal, and reproduction change. Studies on other lepidopteran insects, such as *Spodoptera littoralis* (Lepidoptera: Noctuidae) and *Spodoptera frugiperda* (Lepidoptera: Noctuidae), have shown that bacterial diversity and community structure can change markedly during pupation and adult emergence [22,23]. However, these studies have mainly focused on agricultural pests, whereas forest defoliators with narrower host ranges remain poorly studied. *H. vitessoides* is closely associated with *Aquilaria* trees and causes severe defoliation during the larval stage. This species therefore provides a useful case for examining bacteria associated with whole eggs and gut tissues in a forest lepidopteran pest.

In this study, we used 16S rRNA gene sequencing to characterize bacteria associated with whole eggs and gut tissues from larvae, male and female pupae, and male and female adults of *H. vitessoides*. Because complete metamorphosis involves marked changes in feeding activity, gut structure, and host physiology, we examined whether bacterial richness, diversity, and community composition differed among these sampled groups and whether differences between sexes were evident in pupal or adult gut samples. PICRUSt2 was used to explore broad functional potential, and a preliminary egg surface sterilization assay was conducted to examine whether reducing culturable bacteria on the egg surface was associated with early larval development. Together, these analyses provide a baseline description of bacteria associated with whole eggs and gut tissues of *H. vitessoides* and highlight candidate taxa that can be tested in future functional studies.

## 2. Materials and Methods

### 2.1. Test Insect Source

Larvae of *H. vitessoides* were collected from Qinzhou City, Guangxi Zhuang Autonomous Region, China, between 2023 and 2025 to establish and maintain a laboratory colony. The colony was maintained at 26 ± 2 °C, 60 ± 5% relative humidity, and a 16 h light:8 h dark photoperiod. Larvae were fed fresh leaves of *Aquilaria sinensis* (Thymelaeaceae) collected from the same host-plant source, and emerged adults were supplied with 10% honey water for colony maintenance. All samples used for 16S rRNA gene sequencing were collected from the same laboratory reared cohort under identical rearing conditions to reduce variation among groups caused by batch effects. The egg surface sterilization and developmental observation assay was performed using egg masses obtained from the laboratory colony at multiple sampling periods.

### 2.2. Specimen Collection

Newly hatched first-instar larvae of *H. vitessoides* were reared in clean polypropylene boxes (20 cm × 10 cm × 5 cm), and fresh leaves of *A. sinensis* were provided daily. Larvae were kept in the same rearing boxes until pupation, and pupae were allowed to emerge as adults in these boxes to avoid transfer-related environmental variation. For 16S rRNA gene sequencing, samples were collected from six groups: 1-day-old whole eggs (E), 1-day-old larval guts (L), 3-day-old male pupal guts (MP), 3-day-old female pupal guts (FP), 1-day-old male adult guts (M), and 1-day-old female adult guts (F). Larvae were starved for 24 h before dissection to reduce food residues in the gut. Adults used for sequencing were collected within 24 h after emergence and before feeding. Pupae and adults were sexed based on external morphology, using the genital/anal region in pupae and the terminal abdominal structures in adults. Each group included three pooled biological replicates. Each replicate consisted of 20 pooled guts for larvae, pupae, or adults, whereas each egg replicate consisted of 100 pooled whole eggs. Pooling was used to obtain sufficient DNA, and each pooled sample was treated as one biological replicate. Thus, the pooled sample, rather than the individual insect, was used as the statistical unit. Whole egg samples were used to characterize bacteria associated with whole eggs, whereas larval, pupal, and adult samples were used to characterize bacteria from intact guts. Previous studies have used intact or whole gut samples to assess gut microbial composition in lepidopteran insects [24,25]. Gut dissections were performed under sterile conditions on a clean bench. Intact guts were removed, and attached fat bodies and surrounding tissues were carefully cleared to reduce contamination from non-gut tissues. All samples were homogenized before DNA extraction.

To examine whether reducing culturable bacteria on the egg surface was associated with host development, egg masses were collected 48 h after oviposition and randomly assigned to a surface-sterilized treatment or an untreated control. Based on preliminary tests, eggs in the treatment group were immersed in 1% sodium hypochlorite (Sinopharm Chemical Reagent Co., Ltd., Shanghai, China) for 1 min, washed twice with 75% ethanol (Sinopharm Chemical Reagent Co., Ltd., Shanghai, China) for 30 s each, and rinsed three times with sterile water [26]. Treated eggs were placed on Luria–Bertani (LB) agar plates to check for culturable bacterial growth. Bacterial 16S rRNA gene PCR was also used as an auxiliary check for bacterial DNA detection in untreated eggs, surface sterilized eggs, and the PCR negative control. Only treated egg batches with no visible colonies on LB agar plates and no visible amplification signal in the auxiliary 16S rRNA gene PCR check were used for the developmental observation assay. Newly hatched larvae from both treated and control eggs were then transferred to the same sterile semi-artificial diet. Emerging adults were provided with sterile 10% honey solution. Hatching rate (*n* = 100 eggs per replicate), pupation rate (*n* = 20 larvae per replicate), emergence rate (*n* = 20 pupae per replicate), developmental duration, and adult sex ratio were recorded. Each treatment included three biological replicates, and all insects were reared in identical culture bottles under the same environmental conditions.

### 2.3. DNA Extraction and 16S rRNA Gene Sequencing

Total DNA was extracted from whole eggs and dissected gut samples using the E.Z.N.A.^®^ Soil DNA Kit (Omega Bio-tek, Norcross, GA, USA). DNA purity and concentration were assessed via 1% agarose gel electrophoresis. The variable V3–V4 region of the 16S rRNA gene was amplified using specific primers (338F: 5′-ACTCCTACGGGAGGCAGCAG-3′ and 806R: 5′-GGACTACHVGGGTWTCTAAT-3′) [27]. Primers were tailed with sample-specific barcode sequences to distinguish each sample. Each 20 μL PCR reaction contained 4 μL of 5× FastPfu buffer, 2 μL of 2.5 mM dNTPs, 0.8 μL of forward primer (5 μM), 0.8 μL of reverse primer (5 μM), 0.4 μL of FastPfu DNA Polymerase, 10 ng of template DNA, and DNase-free water. The 5× FastPfu buffer, dNTPs, FastPfu DNA Polymerase, and DNase-free water were obtained from TransGen Biotech Co., Ltd. (Beijing, China), and the primers were synthesized by Sangon Biotech Co., Ltd. (Shanghai, China). The PCR amplification was performed as follows: initial denaturation at 95 °C for 3 min, followed by 27 cycles of denaturing at 95 °C for 30 s, annealing at 60 °C for 30 s and extension at 72 °C for 45 s, with a final extension at 72 °C for 10 min and holding at 4 °C (T100 Thermal Cycler, Bio-Rad, Hercules, CA, USA). After electrophoresis, the PCR products were purified using the AMPure^®^ PB beads (Pacific Biosciences, Menlo Park, CA, USA) and quantified with Qubit 4.0 (Thermo Fisher Scientific, Waltham, MA, USA). Purified amplicons were pooled in equimolar amounts and sequenced on an Illumina NextSeq 2000 platform (Illumina, San Diego, CA, USA) according to the standard protocols of Majorbio Bio-Pharm Technology Co., Ltd. (Shanghai, China).

### 2.4. Data Processing

Raw paired-end reads were first filtered using fastp (version 0.20.0) to remove low-quality sequences and adapter contamination [28]. High-quality reads were merged using FLASH (version 1.2.7) [29]. The merged sequences were imported into QIIME 2 (version 2023.7) [30], and DADA2 was used for denoising, chimera removal, and ASV inference [31]. Taxonomic assignment of ASVs was performed using a naive Bayes classifier trained against the SILVA 16S rRNA gene database [32]. Alpha diversity indices, including ACE, Chao1, Simpson, and Shannon–Wiener indices, were calculated from the ASV table [33]. Venn diagrams and UpSet plots were used to show shared and unique ASVs among groups. Principal coordinate analysis (PCoA) and non-metric multidimensional scaling (NMDS) based on Bray–Curtis dissimilarities were used to compare bacterial community structure. LEfSe analysis was used to identify candidate discriminative bacterial taxa among groups, with an LDA score threshold of 2.0 and *p* < 0.05 [34]. To account for the compositional nature of 16S rRNA gene sequencing data, differential abundance analysis was further performed using ALDEx2 with centered log-ratio transformation [35]. *p* values were adjusted using the Benjamini–Hochberg method, and ASVs with Benjamini–Hochberg-adjusted ALDEx2 GLM *p* values (glm.eBH) < 0.05 were considered candidate differentially abundant ASVs [36]. LEfSe and ALDEx2 were used to identify bacterial taxa associated with differences among groups, and the results were interpreted cautiously. PICRUSt2 was used to predict potential functional profiles based on ASV sequences and the KEGG pathway database [37]. The reliability of PICRUSt2 prediction was evaluated using NSTI values.

### 2.5. Data Analysis

Statistical analyses were performed using JASP (version 0.19.3.0) and R (version 4.4.2). Differences in alpha diversity indices among groups were first tested using Kruskal–Wallis tests. Pairwise Wilcoxon rank sum tests were then performed, and *p* values were adjusted using the Benjamini and Hochberg false discovery rate method [36]. Differences in bacterial community structure based on Bray–Curtis dissimilarities were assessed using ANOSIM with 999 permutations. As an additional test of beta diversity, PERMANOVA was performed using the adonis2 function in the vegan package in R [38,39]. Homogeneity of multivariate dispersion was assessed using betadisper with 9999 permutations. Normally distributed data were compared using one-way ANOVA followed by Tukey’s HSD test. Differences were considered statistically significant at *p* < 0.05. Statistical results were reported using the standard test statistics for each method, including ANOSIM *R*, PERMANOVA *R*^2^ and *F*, betadisper *F*, and corresponding *p* values.

## 3. Results

### 3.1. Summary of Sequencing Data

**Figure 1:**
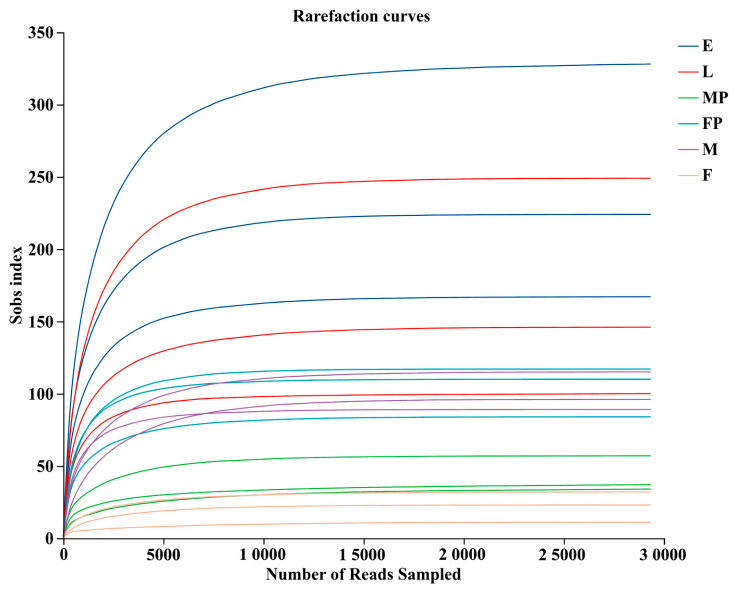
Rarefaction curves of bacterial communities from eggs and gut samples of *H. vitessoides*.

High-throughput sequencing was performed on whole egg samples and gut samples from larvae, male and female pupae, and male and female adults of *H. vitessoides*. After quality filtering and sequence optimization, 935,721 high quality sequences were obtained, with an average sequence length of 424 bp (Table 1). Across the 18 individual samples, the summed number of observed ASV occurrences was 2021. For group-level analyses, ASV IDs were merged within each group, so that ASVs shared by the three replicate samples of a group were counted only once. This produced 1209 ASVs for the downstream Venn and UpSet analyses. Whole egg samples generally contained more observed ASVs, whereas female adult gut samples showed the lowest ASV richness. Rarefaction curves approached saturation, indicating that the sequencing depth was sufficient to capture the bacterial diversity of the analyzed samples (Figure 1).

### 3.2. Bacterial Diversity Across Developmental Stages and Sexes

**Figure 2:**
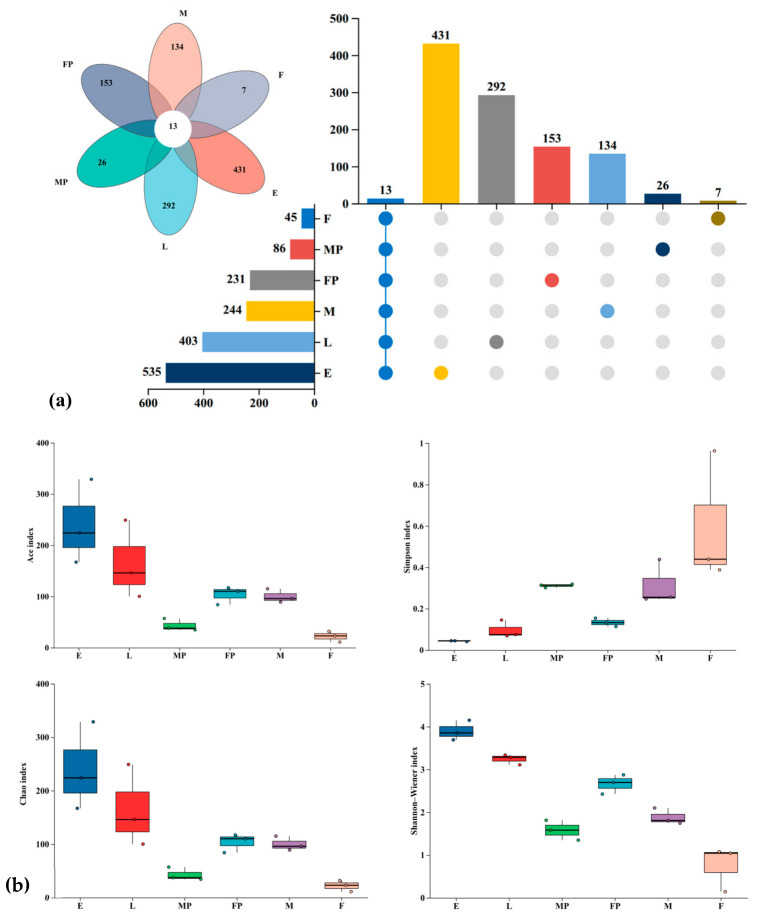
Shared ASVs and alpha diversity of bacterial communities in whole egg and gut samples of *H. vitessoides*. (**a**) Venn and UpSet analyses showing shared and unique ASVs among whole eggs, larval guts, male pupal guts, female pupal guts, male adult guts, and female adult guts. (**b**) Alpha diversity indices, including ACE, Chao1, Simpson, and Shannon–Wiener indices. Group-level summaries and statistical test results for the alpha-diversity indices are provided in Table S1. In the UpSet plot, filled dots and connecting lines indicate ASV intersections among groups, and vertical bars show intersection sizes.

Based on the 1209 ASVs obtained after merging replicate samples within each group, Venn and UpSet analyses showed shared and unique ASVs among whole eggs, larval guts, male pupal guts, female pupal guts, male adult guts, and female adult guts (Figure 2a). Whole egg samples contained the largest number of unique ASVs, with 431 unique ASVs, whereas female adult gut samples contained the fewest unique ASVs, with only seven unique ASVs. Only 13 ASVs were shared across all groups, suggesting that most detected ASVs were restricted to particular sampled groups. Group-level summaries and statistical test results for the alpha-diversity indices are provided in Table S1. Whole egg samples and larval gut samples generally showed higher richness and diversity values than pupal and adult gut samples (Figure 2b). Kruskal–Wallis tests indicated overall differences in alpha diversity indices among groups, whereas pairwise Wilcoxon rank sum tests did not detect significant differences after correction using the Benjamini and Hochberg method. Therefore, these alpha diversity patterns were interpreted as overall group level trends rather than strong pairwise differences.

### 3.3. Dynamics of Bacterial Community Composition

**Figure 3:**
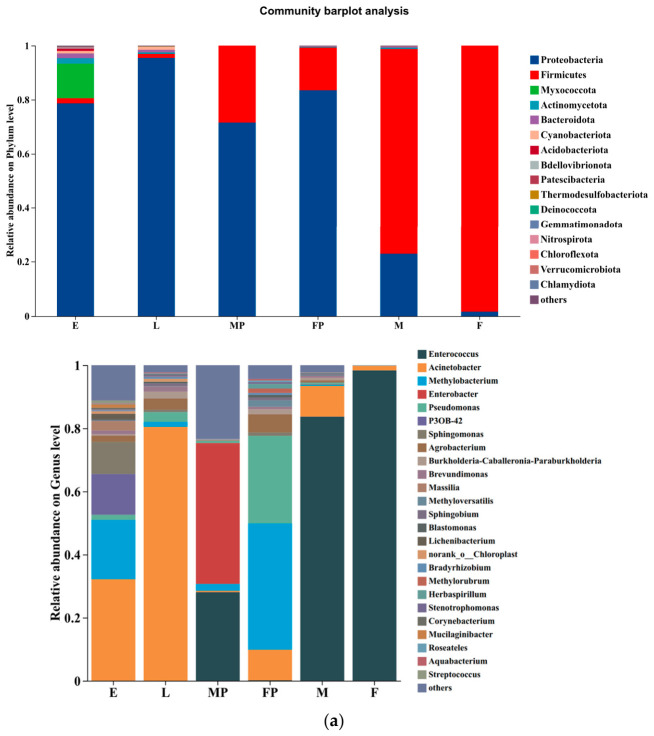

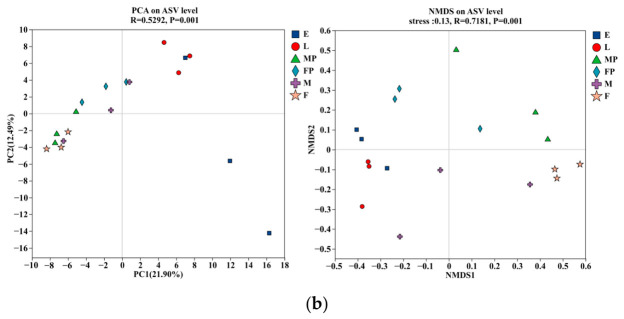
Bacterial community composition and beta diversity patterns in whole egg and gut samples of *H. vitessoides*. (**a**) Relative abundance of bacterial taxa at the phylum and genus levels. (**b**) PCoA and NMDS based on Bray–Curtis dissimilarities. PERMANOVA and betadisper results are summarized in Table S2.

At the phylum level, bacterial composition differed among the six sampled groups (Figure 3a). Female adult gut samples were dominated by Firmicutes, with a relative abundance approaching 100%. In contrast, larval gut samples were dominated by Proteobacteria, accounting for more than 90% of the community. In female pupal gut samples, Proteobacteria accounted for more than 80%, with Firmicutes as the secondary phylum. In male pupal gut samples, Proteobacteria and Firmicutes had similar proportions, forming a dual dominant pattern. Male adult gut samples were dominated by Firmicutes, whereas Proteobacteria accounted for approximately 20%. Whole egg samples contained a broader set of secondary phyla, including Myxococcota and Actinomycetota. At the genus level, dominant taxa differed among groups. Male pupal gut samples were mainly composed of *Enterococcus* and *Enterobacter*. Whole egg samples showed the highest genus level complexity, with multiple genera, including *Methylobacterium* and *Sphingomonas*, coexisting. Larval gut samples were dominated by *Acinetobacter*, whereas *Enterococcus* was highly abundant in both male and female adult gut samples.

PCoA and NMDS based on Bray–Curtis dissimilarities showed separation among the sampled groups (Figure 3b). Whole egg samples, larval gut samples, pupal gut samples, and adult gut samples formed distinct clusters, indicating differences in bacterial community composition among the groups examined in this study. Sex related separation was most evident in pupal gut samples, whereas male and female adult gut samples were more similar. ANOSIM supported differences among groups (*R* = 0.650, *p* = 0.001). PERMANOVA further showed significant differences among the six sampled groups (adonis2, *R*^2^ = 0.533, *F* = 2.738, *p* = 0.0001; Table S2), and betadisper was not significant (*F* = 0.637, *p* = 0.673). To reduce the influence of the difference between whole eggs and gut tissues, we also performed a separate analysis of gut samples after excluding egg samples. This analysis showed significant differences among the larval, pupal, and adult gut bacterial communities (adonis2, *R*^2^ = 0.496, *F* = 2.458, *p* = 0.0016), with no significant difference in multivariate dispersion among the gut sample groups (betadisper, *F* = 0.729, *p* = 0.588; Table S2).

### 3.4. Relative Abundance Patterns of Dominant Bacterial Genera

**Figure 4:**
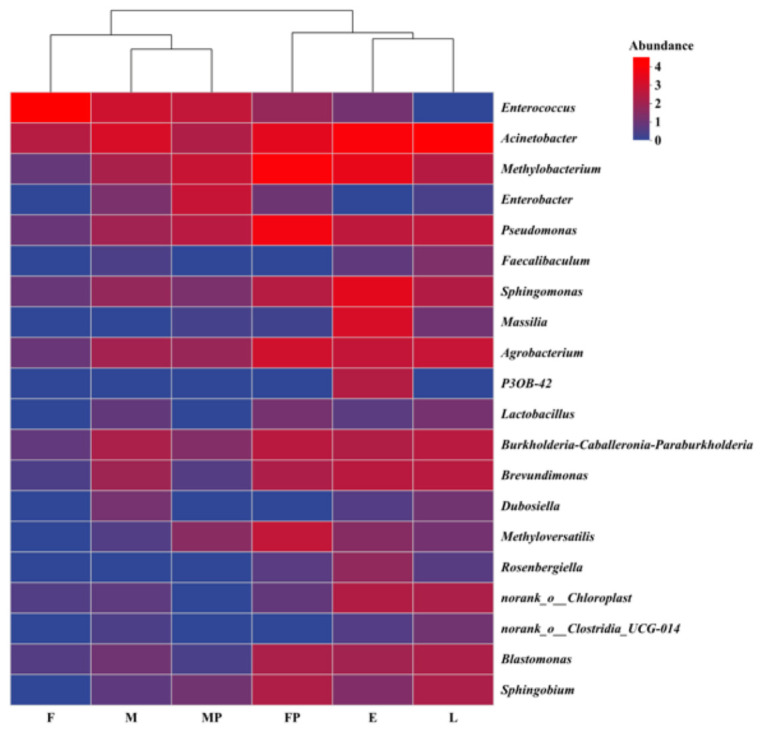
Heatmap showing the relative abundance of dominant bacterial genera among the six sampled groups of *H. vitessoides*.

Proteobacteria and Firmicutes were the dominant bacterial phyla across the sampled groups, but their relative abundances differed among whole egg and gut samples (Figure 4). Proteobacteria were more abundant in whole egg and larval gut samples, whereas Firmicutes became more abundant in pupal and adult gut samples. At the genus level, several dominant taxa showed clear differences among groups. *Acinetobacter* was highly abundant in larval gut samples and was also detected in whole egg samples. *Enterococcus* showed high relative abundance in adult gut samples, especially in female adults. *Pseudomonas* and *Methylobacterium* were relatively abundant in female pupal gut samples, whereas *Sphingomonas* and *Massilia* were more common in whole egg samples. These patterns indicate that bacterial composition differed among the sampled groups, with differences between sexes being more evident in the pupal gut samples.

**Figure 5:**
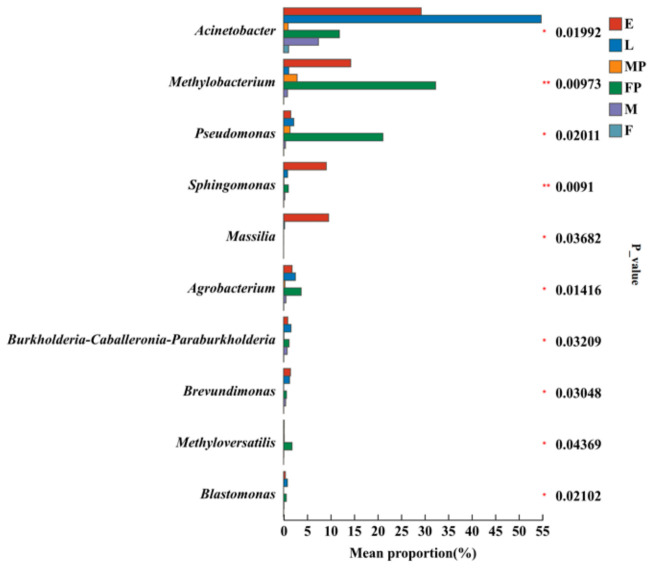
Relative abundance patterns of selected dominant bacterial genera among the six sampled groups of *H. vitessoides* based on Kruskal–Wallis tests. Asterisks indicate the significance levels of differences among groups based on Kruskal–Wallis tests (*, *p* < 0.05; **, *p* < 0.01).

Kruskal–Wallis tests were used to examine differences in the relative abundance of selected dominant genera among groups (Figure 5). *Acinetobacter* accounted for more than 50% of the bacterial community in larval gut samples (*p* < 0.05). *Methylobacterium* accounted for nearly 30% of the community in female pupal gut samples (*p* < 0.01), whereas *Pseudomonas* accounted for approximately 20% in the same group (*p* < 0.05). *Sphingomonas* and *Massilia* showed higher relative abundances in whole egg samples (*p* < 0.05). *Methyloversatilis* was also more abundant in female pupal gut samples (*p* < 0.05). These genera were therefore treated as dominant taxa showing differences among groups, rather than confirmed functional bacterial markers.

### 3.5. Candidate Discriminative Bacterial Taxa Across Groups

**Figure 6:**
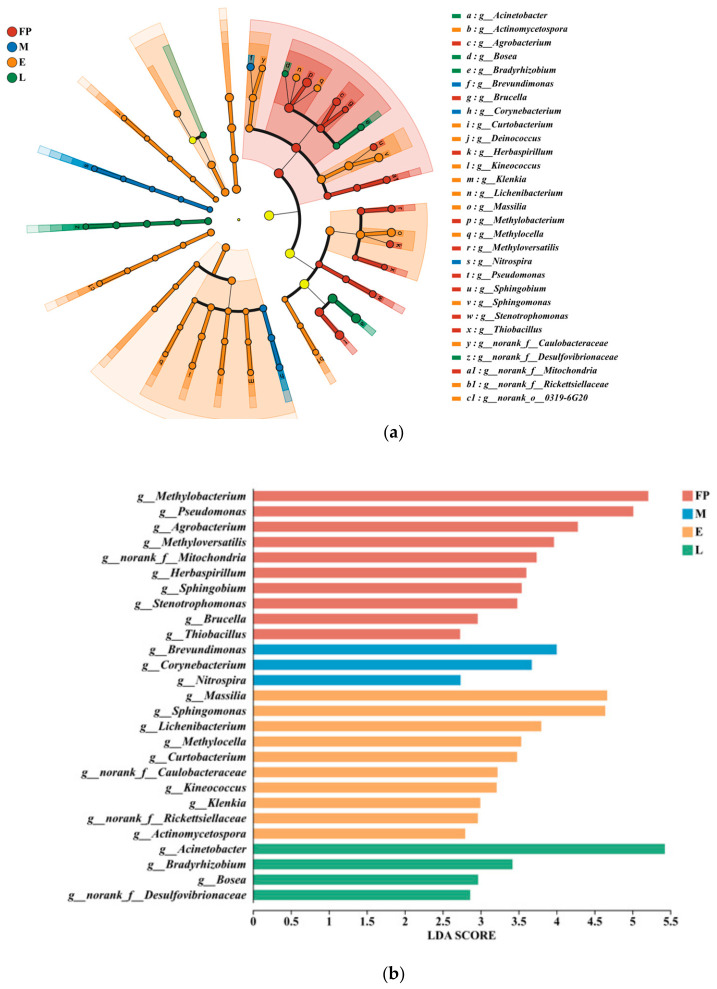
LEfSe analysis of candidate discriminative bacterial taxa among the six sampled groups of *H. vitessoides*. Only groups with discriminative taxa are shown. (**a**) Cladogram showing candidate taxa associated with different groups. (**b**) LDA score histogram showing discriminative taxa among groups.

LEfSe analysis was used to identify candidate bacterial taxa that contributed to group separation (Figure 6). *Acinetobacter* showed high relative abundance in larval gut samples and was the main taxon associated with the larval group in the LEfSe analysis. *Bradyrhizobium* and *Agrobacterium* were mainly associated with whole egg samples, whereas *Sphingomonas* and *Brevundimonas* were enriched in female pupal gut samples. Other genera, including *Methylobacterium*, *Pseudomonas*, *Blastococcus*, and *Rhodococcus*, also showed enrichment in particular groups. These taxa were treated as candidate discriminative genera.

As a complementary analysis, ALDEx2 was used to account for the compositional nature of 16S rRNA gene sequencing data. The GLM based ALDEx2 analysis identified 26 candidate differentially abundant ASVs after correction using the Benjamini and Hochberg method (glm.eBH < 0.05). These candidate ASVs were mainly assigned to *Acinetobacter*, *Methylobacterium*, *Brevundimonas*, *Pseudomonas*, *Sphingobium*, and other genera, and their highest mean relative abundances were observed mainly in larval gut, whole egg, and female pupal gut samples (Table S3). Together, LEfSe and ALDEx2 were used to identify candidate taxa associated with differences among groups, rather than confirmed bacterial biomarkers.

### 3.6. Functional Prediction of Bacterial Communities in H. vitessoides

**Figure 7:**
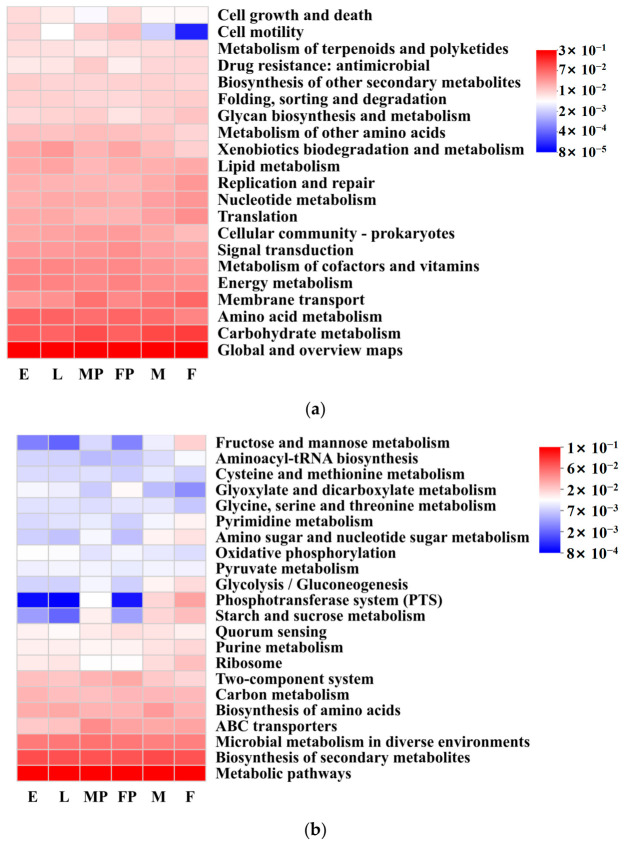
Predicted KEGG functional profiles of bacterial communities associated with whole eggs and gut tissues of *H. vitessoides*. (**a**) Relative abundance of predicted KEGG pathways at level 2. (**b**) Relative abundance of predicted KEGG pathways at level 3. NSTI values used to evaluate PICRUSt2 prediction reliability are summarized in Table S4.

PICRUSt2 was used to predict potential functional profiles of bacterial communities associated with whole eggs and gut tissues of *H. vitessoides*. At KEGG pathway level 2, the most abundant predicted functional categories included global and overview maps, carbohydrate metabolism, amino acid metabolism, membrane transport, energy metabolism, metabolism of cofactors and vitamins, and signal transduction (Figure 7a). At KEGG pathway level 3, the dominant predicted pathways included metabolic pathways, biosynthesis of secondary metabolites, and microbial metabolism in diverse environments (Figure 7b). These predicted profiles were mainly related to broad metabolic and transport functions. Because PICRUSt2 predictions are inferred from 16S rRNA gene profiles, these results should be interpreted as functional potential rather than direct evidence of gene expression or metabolic activity.

The reliability of PICRUSt2 prediction was assessed using NSTI values. The final NSTI table included 1209 ASVs and matched the unique ASV table used for downstream analyses. The median ASV level NSTI value was 0.095, with an interquartile range of 0.035–0.211. In addition, 72.5% of ASVs had NSTI values ≤ 0.20, and 92.5% had NSTI values ≤ 0.50. Abundance weighted NSTI values were calculated for each sample and summarized by group; group level values ranged from 0.038 ± 0.000 in male adult gut samples to 0.113 ± 0.045 in whole egg samples (Table S4). These results indicate that the predicted profiles were mainly based on taxa with relatively close reference genomes, although a small proportion of ASVs had high NSTI values. The PICRUSt2 results were therefore treated as exploratory functional predictions.

### 3.7. Association Between Egg Surface Sterilization and Developmental Duration

**Figure 8:**
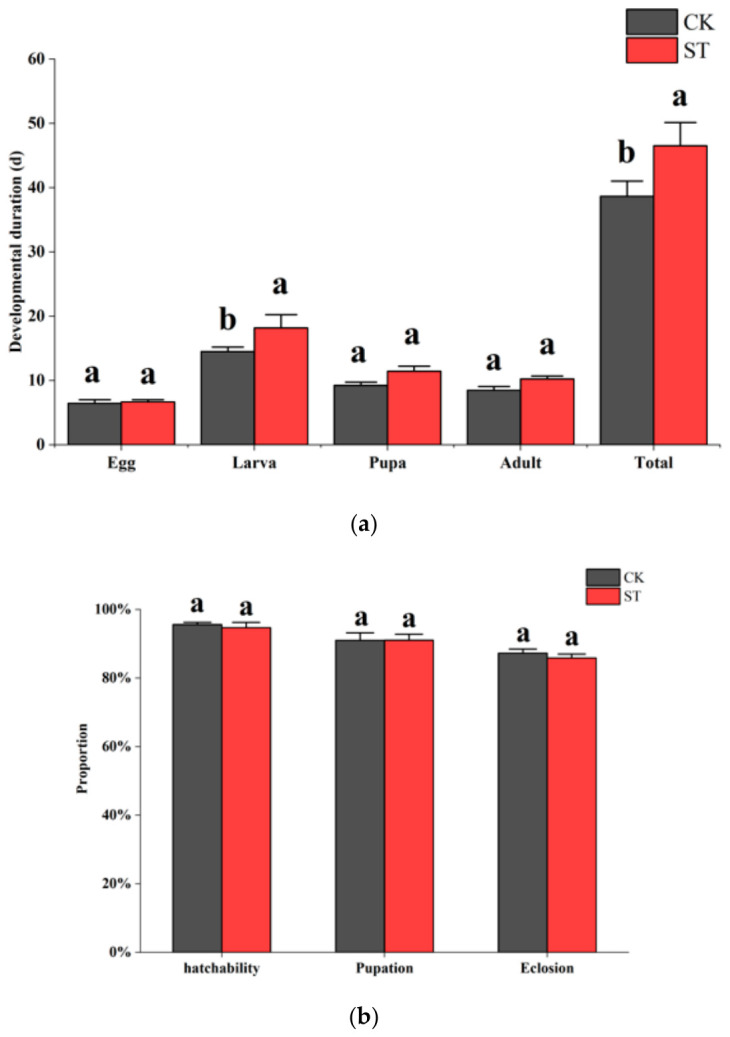

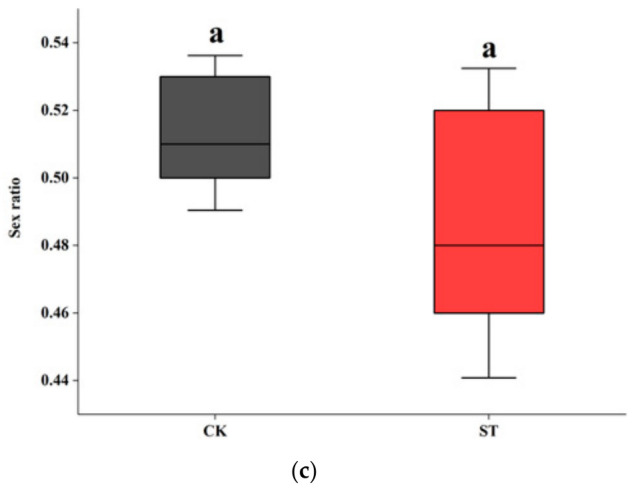
Association between egg surface sterilization and developmental traits of *H. vitessoides*. (**a**) Developmental duration of the untreated control group (CK) and surface-sterilized treatment group (ST). (**b**) Hatching, pupation, and emergence rates. (**c**) Adult sex ratio. Error bars represent ± SE. Different lowercase letters indicate significant differences between treatments within the same developmental stage or trait at *p* < 0.05.

Egg surface sterilization was followed by a longer larval developmental period (Figure 8a). Larvae from treated eggs required more time to develop (18.17 ± 2.07 d) than larvae from untreated control eggs (14.50 ± 0.72 d; *p* < 0.05), and total developmental duration was also significantly longer in the treated group (*p* < 0.05). In contrast, egg, pupal, and adult developmental durations did not differ significantly between the two groups. Hatching rate, pupation rate, emergence rate, and adult sex ratio also showed no significant differences between the treated and control groups (Figure 8b,c). These results indicate a preliminary association between egg surface sterilization and delayed larval development, with the delay contributing to an extended total developmental duration, although the role of egg associated bacteria and the contribution of specific bacterial taxa require further testing.

## 4. Discussion

This study showed that bacterial richness, diversity, and community composition differed among whole egg samples and gut samples from the larvae, pupae, and adults of *H. vitessoides*. Whole egg and larval gut samples generally contained richer bacterial communities than the pupal and adult gut samples. This pattern is consistent with a reduction or reorganization in bacterial communities after the actively feeding larval stage, although the whole egg samples and later gut samples represent different biological sample types. Comparable shifts have been reported in other lepidopteran insects, but the direction and dominant taxa are not identical among species. In *Spodoptera littoralis* and *Brithys crini* (Lepidoptera: Noctuidae), gut bacterial communities changed across metamorphosis, indicating that pupation and adult emergence can act as major transition points for insect associated bacteria [22,40]. In *Grapholita molesta* (Lepidoptera: Tortricidae), the dominant genera shifted from *Gluconobacter* and *Pantoea* in early larvae to *Enterococcus* and *Enterobacter* in late larvae and to *Serratia* in pupae [41]. In the potato tuber moth, *Enterococcus mundtii* was rare in eggs and first instar larvae, became highly abundant in later larvae and pupae, and then declined in adults [42]. In contrast, *H. vitessoides* showed high bacterial richness in whole egg and larval gut samples, a larval gut community dominated by *Acinetobacter*, and adult gut communities dominated by *Enterococcus*. These differences may reflect differences in host species, diet, gut remodeling during metamorphosis, adult feeding status, and the fact that our egg samples represented whole eggs whereas later samples represented dissected guts.

The relatively high number of ASVs in whole egg samples may reflect several bacterial sources, including maternal contact, the egg surface, host plants, the rearing substrate, and the surrounding environment. A study comparing lepidopteran eggs, larvae, adults, and host plant materials showed that insect associated bacterial communities can differ among developmental stages while also being linked to the surrounding plant environment [43]. Maternal and environmental contributions to early bacterial communities have also been discussed in Lepidoptera and other holometabolous insects [17,19]. The occurrence of plant associated or environment associated genera such as *Methylobacterium*, *Sphingomonas*, and *Agrobacterium* in whole egg samples is consistent with these possible sources, but it should not be taken as evidence that these bacteria were located inside the eggs. Larval guts also showed relatively high bacterial richness, which may reflect active feeding on fresh *Aquilaria* leaves and repeated exposure to plant associated and environmental bacteria during feeding [44,45]. Diet can influence the establishment, proliferation, and stability of gut bacterial populations in herbivorous lepidopteran larvae [46]. The high relative abundance of *Acinetobacter* in larval guts may therefore be related to the larval feeding environment or to bacteria introduced with plant material. Some gut bacteria of lepidopteran herbivores have been linked to the processing of plant defensive compounds, but whether *Acinetobacter* or other dominant bacteria contribute to digestion, detoxification, or larval growth in *H. vitessoides* remains to be tested [47,48,49].

Proteobacteria and Firmicutes were the dominant phyla in *H. vitessoides*. These two phyla are frequently reported as major components of lepidopteran gut bacterial communities, although their relative abundances can vary with host species, diet, and developmental stage [22,23]. In the present study, Proteobacteria were more abundant in whole egg and larval gut samples, whereas Firmicutes became dominant in adult gut samples. Adult gut samples were dominated by Firmicutes, particularly *Enterococcus*. Previous studies have reported that *Enterococcus* can be abundant in lepidopteran insects feeding on chemically defended host plants and may also be linked to host physiological metabolism [50]. In *Galleria mellonella* (Lepidoptera: Pyralidae), intestinal *Enterococcus* populations changed during metamorphosis, suggesting that this genus may respond to major shifts in gut conditions [51]. These studies provide useful comparisons for the high abundance of *Enterococcus* in adult *H. vitessoides* guts, but they do not demonstrate the same function in this species. In our study, we did not test whether *Enterococcus* contributes to adult physiology, nutrient use, or reproduction. Its dominance in adult gut samples should therefore be interpreted as a community pattern rather than evidence of a defined function.

Differences between sexes were more evident in pupal gut samples than in adult gut samples. Male pupal guts were associated with *Enterococcus* and *Enterobacter*, whereas female pupal guts showed higher relative abundances of *Methylobacterium* and *Pseudomonas*. Similar differences between sexes have been reported in other lepidopteran insects. For example, studies on *S*. *frugiperda* and *G*. *molesta* found that male and female pupae or adults differed in bacterial community composition or dominant genera [23,41]. In contrast, work on *Plutella xylostella* (Lepidoptera: Plutellidae) suggests that developmental stage may have a stronger effect on bacterial community structure than sex or infection status in some systems [52]. In the present study, male and female adult gut samples were more similar than pupal gut samples, suggesting that differences between sexes in *H. vitessoides* may vary between pupal and adult stages rather than being consistent after emergence.

PICRUSt2 predicted functional categories mainly related to carbohydrate metabolism, amino acid metabolism, energy metabolism, membrane transport, and cofactor and vitamin metabolism in bacterial communities associated with *H. vitessoides*. Similar functional categories have been predicted in other phytophagous insects and lepidopteran species [53,54]. These similarities indicate that the predicted profiles of bacteria associated with *H. vitessoides* fall within functional categories commonly inferred from herbivorous insect microbiomes. Such categories are biologically plausible in the context of larval feeding, because plant leaves are nutritionally unbalanced and contain complex carbohydrates and defensive compounds [55]. However, these results should not be interpreted as evidence that the bacteria detected here directly contribute to nutrient acquisition, plant food use, or host development. This point is especially important because PICRUSt2 infers functional potential from 16S rRNA gene profiles rather than measuring gene content, gene expression, enzyme activity, or metabolites [37]. In this study, NSTI values were added to evaluate the reliability of PICRUSt2 prediction. The median NSTI value across ASVs was 0.095, and abundance weighted NSTI values were low overall, suggesting that most predictions were based on taxa with relatively close reference genomes. Even so, the PICRUSt2 results should be viewed as exploratory hypotheses. Metagenomic, transcriptomic, metabolomic, isolation, and reinoculation studies will be needed to test the functions of specific bacterial taxa.

The egg surface sterilization assay showed that larvae from treated eggs developed more slowly than larvae from untreated controls, and this delay was accompanied by a longer total developmental duration. In contrast, hatching rate, pupation rate, emergence rate, and adult sex ratio did not differ significantly between treatments. This result suggests a link between egg surface treatment and larval developmental duration, but it does not demonstrate that egg associated bacteria directly caused the delay. Similar links between insect associated bacteria and host development have been reported in other systems [56], and the gut bacteria of lepidopteran herbivores have been linked to the processing of plant defensive compounds [47]. Therefore, this assay should be interpreted as preliminary evidence of an association rather than functional evidence for a specific bacterial taxon. Future work should combine bacterial load measurements, 16S rRNA gene profiling of treated and untreated eggs, bacterial isolation, and reinoculation to test whether particular egg associated or early colonizing bacteria affect larval development in *H. vitessoides*.

## 5. Conclusions

This study characterized bacteria associated with whole eggs and gut tissues from the larvae, pupae, and adults of *H. vitessoides*. Whole egg and larval gut samples generally harbored richer bacterial communities than pupal and adult gut samples, and bacterial composition differed among the six sampled groups. Proteobacteria were more common in whole egg and larval gut samples, whereas Firmicutes, particularly *Enterococcus*, dominated the adult gut samples. Sex related differences were more evident in pupal gut samples than in adult gut samples. PICRUSt2 predicted broad functional categories related mainly to nutrient metabolism and membrane transport, but these predictions should be regarded as functional potential. The egg surface sterilization assay was associated with longer larval and total developmental durations, suggesting a possible link between early egg surface treatment and larval development. Overall, these findings provide baseline information on egg associated and gut bacteria of *H. vitessoides* and identify candidate bacterial taxa for future functional studies.

## Figures and Tables

**Table 1:** Summary of the 16S rRNA gene sequencing data for bacterial communities associated with whole eggs and gut tissues of *H. vitessoides*.

Samples	Valid Reads	Average Length (bp)	Min Length (bp)	Max Length (bp)	Number of ASVs
E	57,474 ± 8481	419.7 ± 3.5	212 ± 6	464 ± 40	240 ± 82
L	57,619 ± 13,903	425.0 ± 1.7	219 ± 19	488 ± 46	165 ± 76
MP	49,119 ± 6613	428.7 ± 1.5	278 ± 109	462 ± 51	43 ± 12
FP	45,237 ± 4220	418.3 ± 1.5	143 ± 77	526 ± 4	104 ± 17
M	48,137 ± 1981	430.0 ± 0.0	231 ± 5	483 ± 44	100 ± 13
F	54,322 ± 9922	427.3 ± 2.1	205 ± 8	517 ± 10	22 ± 11

Values for sequences, average length, and observed ASVs are shown as mean ± SD.

## Data Availability

The sequencing data will be available in the NCBI Sequence Read Archive under BioProject accession number PRJNA1369093 upon publication.

## Supplementary Materials

The following supporting information can be downloaded at: https://www.mdpi.com/article/10.3390/insects17070668/s1, Table S1. Alpha diversity summaries and statistical tests among sampled groups; Table S2. PERMANOVA and betadisper results based on Bray–Curtis dissimilarities; Table S3. Candidate differentially abundant ASVs identified by ALDEx2 and their taxonomic annotations; Table S4. NSTI values used to evaluate PICRUSt2 functional prediction.

## References

[1] Kuntadi K., Irianto R.S., Andadari L. (2016). Dinamika Serangan Ulat *Heortia vitessoides* Moore (Lepidoptera: Crambidae) Pada Tanaman Gaharu Di Hutan Penelitian Carita, Propinsi Banten. J. Penelit. Hutan Tanam..

[2] Prathapan K.D., Santhoshkumar T. (2023). First Report of Infestation of the Agar Defoliator *Heortia vitessoides* (Moore) on the Agar Wood Tree *Aquilaria malaccensis* Benth. in South India. Indian J. Entomol..

[3] Yin Z., Chen Y., Xue H., Li X., Li B., Liang J., Zhu Y., Long K., Yang J., Pang J. (2025). *Heortia vitessoides* Infests *Aquilaria sinensis*: A Systematic Review of Climate Drivers, Management Strategies, and Molecular Mechanisms. Insects.

[4] Xu D., Li X., Jin Y., Zhuo Z., Yang H., Hu J., Wang R. (2020). Influence of Climatic Factors on the Potential Distribution of Pest *Heortia vitessoides* Moore in China. Glob. Ecol. Conserv..

[5] Chen Z., Li D., Wang L., Li Y., Huang X., Qin C. (2011). Studies on Biological Characteristics of *Heortia vitessoides* Moore on *Aquilaria sinensis*. China Plant Prot..

[6] National Pharmacopoeia Commission (2015). Pharmacopoeia of the People’s Republic of China.

[7] Jin X., Ma T., Chang M., Wu Y., Liu Z., Sun Z., Shan T., Chen X., Wen X., Wang C. (2016). Aggregation and Feeding Preference of Gregarious *Heortia vitessoides* (Lepidoptera: Crambidae) Larvae to *Aquilaria sinensis* (Thymelaeaceae). J. Entomol. Sci..

[8] Qiao H., Lu P., Chen J., Xu C., Ma W., Qin R., Li X., Cheng H. (2013). Biological Characteristics and Occurrence Patterns of *Heortia vitessoides*. Chin. J. Appl. Entomol..

[9] Chen Y., Hong R., Rao D., Han Y., Chen G., Dong X. (2023). Insect-Resistance of *Aquilaria sinensis* (Lour.) Leaves Is Associated with Volatile Compounds. Russ. J. Plant Physiol..

[10] Han S., Akhtar M.R., Xia X. (2024). Functions and Regulations of Insect Gut Bacteria. Pest Manag. Sci..

[11] Zhang X., Zhang F., Lu X. (2022). Diversity and Functional Roles of the Gut Microbiota in Lepidopteran Insects. Microorganisms.

[12] Basit A., Haq I.U., Hyder M., Humza M., Younas M., Akhtar M.R., Ghafar M.A., Liu T.-X., Hou Y. (2025). Microbial Symbiosis in Lepidoptera: Analyzing the Gut Microbiota for Sustainable Pest Management. Biology.

[13] Cornwallis C.K., van ’t Padje A., Ellers J., Klein M., Jackson R., Kiers E.T., West S.A., Henry L.M. (2023). Symbioses Shape Feeding Niches and Diversification across Insects. Nat. Ecol. Evol..

[14] Zhang S., Song F., Wang J., Li X., Zhang Y., Zhou W., Xu L. (2024). Gut Microbiota Facilitate Adaptation of Invasive Moths to New Host Plants. ISME J..

[15] Kucuk R.A. (2020). Gut Bacteria in the Holometabola: A Review of Obligate and Facultative Symbionts. J. Insect Sci..

[16] Chen L., He Z., Zhang D., Zhao F., Zhang Y., Ding R. (2025). The Role of Gut Microbiota at Different Developmental Stages in the Adaptation of the *Etiella zinckenella* to a Plant Host. Sci. Rep..

[17] Paniagua Voirol L.R., Frago E., Kaltenpoth M., Hilker M., Fatouros N.E. (2018). Bacterial Symbionts in Lepidoptera: Their Diversity, Transmission, and Impact on the Host. Front. Microbiol..

[18] Manthey C., Johnston P.R., Nakagawa S., Rolff J. (2023). Complete Metamorphosis and Microbiota Turnover in Insects. Mol. Ecol..

[19] Jose P.A., Yuval B., Jurkevitch E. (2023). Maternal and Host Effects Mediate the Adaptive Expansion and Contraction of the Microbiome during Ontogeny in a Holometabolous, Polyphagous Insect. Funct. Ecol..

[20] Manthey C., Metcalf C.J.E., Monaghan M.T., Steiner U.K., Rolff J. (2024). Rapid Growth and the Evolution of Complete Metamorphosis in Insects. Proc. Natl. Acad. Sci. USA.

[21] Oishi S., Moriyama M., Mizutani M., Futahashi R., Fukatsu T. (2023). Regulation and Remodeling of Microbial Symbiosis in Insect Metamorphosis. Proc. Natl. Acad. Sci. USA.

[22] Chen B., Teh B.-S., Sun C., Hu S., Lu X., Boland W., Shao Y. (2016). Biodiversity and Activity of the Gut Microbiota across the Life History of the Insect Herbivore *Spodoptera littoralis*. Sci. Rep..

[23] Chen J., Ma Y., Huang S., Li J., Zhang Y., Wang H., Qi G., Shi Q., Zhang Z., Yang M. (2023). The Dynamics of the Microbial Community in Fall Armyworm *Spodoptera frugiperda* during a Life Cycle. Entomol. Exp. Appl..

[24] Jeon J., Rahman M.-M., Han C., Shin J., Sa K.J., Kim J. (2023). *Spodoptera frugiperda* (Lepidoptera: Noctuidae) Life Table Comparisons and Gut Microbiome Analysis Reared on Corn Varieties. Insects.

[25] Zhang N., He J., Shen X., Sun C., Muhammad A., Shao Y. (2021). Contribution of Sample Processing to Gut Microbiome Analysis in the Model Lepidoptera, Silkworm *Bombyx mori*. Comput. Struct. Biotechnol. J..

[26] Mason C.J., Ray S., Shikano I., Peiffer M., Jones A.G., Luthe D.S., Hoover K., Felton G.W. (2019). Plant Defenses Interact with Insect Enteric Bacteria by Initiating a Leaky Gut Syndrome. Proc. Natl. Acad. Sci. USA.

[27] Weisburg W.G., Barns S.M., Pelletier D.A., Lane D.J. (1991). 16S Ribosomal DNA Amplification for Phylogenetic Study. J. Bacteriol..

[28] Chen S., Zhou Y., Chen Y., Gu J. (2018). Fastp: An Ultra-Fast All-in-One FASTQ Preprocessor. Bioinformatics.

[29] Magoč T., Salzberg S.L. (2011). FLASH: Fast Length Adjustment of Short Reads to Improve Genome Assemblies. Bioinformatics.

[30] Bolyen E., Rideout J.R., Dillon M.R., Bokulich N.A., Abnet C.C., Al-Ghalith G.A., Alexander H., Alm E.J., Arumugam M., Asnicar F. (2019). Reproducible, Interactive, Scalable and Extensible Microbiome Data Science Using QIIME 2. Nat. Biotechnol..

[31] Callahan B.J., McMurdie P.J., Rosen M.J., Han A.W., Johnson A.J.A., Holmes S.P. (2016). DADA2: High-Resolution Sample Inference from Illumina Amplicon Data. Nat. Methods.

[32] Quast C., Pruesse E., Yilmaz P., Gerken J., Schweer T., Yarza P., Peplies J., Glöckner F.O. (2013). The SILVA Ribosomal RNA Gene Database Project: Improved Data Processing and Web-Based Tools. Nucleic Acids Res..

[33] Schloss P.D., Westcott S.L., Ryabin T., Hall J.R., Hartmann M., Hollister E.B., Lesniewski R.A., Oakley B.B., Parks D.H., Robinson C.J. (2009). Introducing Mothur: Open-Source, Platform-Independent, Community-Supported Software for Describing and Comparing Microbial Communities. Appl. Environ. Microbiol..

[34] Segata N., Izard J., Waldron L., Gevers D., Miropolsky L., Garrett W.S., Huttenhower C. (2011). Metagenomic Biomarker Discovery and Explanation. Genome Biol..

[35] Fernandes A.D., Reid J.N.S., Macklaim J.M., McMurrough T.A., Edgell D.R., Gloor G.B. (2014). Unifying the Analysis of High-Throughput Sequencing Datasets: Characterizing RNA-Seq, 16S rRNA Gene Sequencing and Selective Growth Experiments by Compositional Data Analysis. Microbiome.

[36] Benjamini Y., Hochberg Y. (1995). Controlling the False Discovery Rate: A Practical and Powerful Approach to Multiple Testing. J. R. Stat. Soc. Ser. B Methodol..

[37] Douglas G.M., Maffei V.J., Zaneveld J.R., Yurgel S.N., Brown J.R., Taylor C.M., Huttenhower C., Langille M.G.I. (2020). PICRUSt2 for Prediction of Metagenome Functions. Nat. Biotechnol..

[38] Oksanen J., Simpson G.L., Blanchet F.G., Kindt R., Legendre P., Minchin P.R., O’Hara R.B., Solymos P., Stevens M.H.H., Szoecs E. (2022). Vegan: Community Ecology Package.

[39] Anderson M.J. (2001). A New Method for Non-Parametric Multivariate Analysis of Variance. Austral Ecol..

[40] González-Serrano F., Pérez-Cobas A.E., Rosas T., Baixeras J., Latorre A., Moya A. (2020). The Gut Microbiota Composition of the Moth *Brithys crini* Reflects Insect Metamorphosis. Microb. Ecol..

[41] Wang X., Sun S., Yang X., Cheng J., Wei H., Li Z., Michaud J.P., Liu X. (2020). Variability of Gut Microbiota Across the Life Cycle of *Grapholita molesta* (Lepidoptera: Tortricidae). Front. Microbiol..

[42] Fu Q., Wang W., Chen B., Hu Y., Ma R., Zhu E., Jin S., Cai H., Xiao G., Du G. (2025). Longitudinal Dynamics of Intestinal Bacteria in the Life Cycle and Their Effects on Growth and Development of Potato Tuber Moth. Front. Microbiol..

[43] Ayayee P.A., Currie A., Peterson J.A. (2022). Different Gut Microbiomes of Developmental Stages of Field-Collected Native and Invasive Western Bean Cutworm, *Striacosta albicosta*, in Western Nebraska. Microorganisms.

[44] Bashir I., War A.F., Rafiq I., Reshi Z.A., Rashid I., Shouche Y.S. (2022). Phyllosphere Microbiome: Diversity and Functions. Microbiol. Res..

[45] Wang Y.-P., Liu X., Yi C.-Y., Chen X.-Y., Liu C.-H., Zhang C.-C., Chen Q.-D., Chen S., Liu H.-L., Pu D.-Q. (2023). The Adaptive Evolution in the Fall Armyworm *Spodoptera frugiperda* (Lepidoptera: Noctuidae) Revealed by the Diversity of Larval Gut Bacteria. Genes.

[46] Mason C.J., St. Clair A., Peiffer M., Gomez E., Jones A.G., Felton G.W., Hoover K. (2020). Diet Influences Proliferation and Stability of Gut Bacterial Populations in Herbivorous Lepidopteran Larvae. PLoS ONE.

[47] Zhang N., Qian Z., He J., Shen X., Lei X., Sun C., Fan J., Felton G.W., Shao Y. (2024). Gut Bacteria of Lepidopteran Herbivores Facilitate Digestion of Plant Toxins. Proc. Natl. Acad. Sci. USA.

[48] Yang Z.L., Seitz F., Grabe V., Nietzsche S., Richter A., Reichelt M., Beutel R., Beran F. (2022). Rapid and Selective Absorption of Plant Defense Compounds From the Gut of a Sequestering Insect. Front. Physiol..

[49] Yuan S., Sun Y., Chang W., Zhang J., Sang J., Zhao J., Song M., Qiao Y., Zhang C., Zhu M. (2023). The Silkworm (*Bombyx mori*) Gut Microbiota Is Involved in Metabolic Detoxification by Glucosylation of Plant Toxins. Commun. Biol..

[50] Fu W., Wang P., He P., Chu D. (2025). Distinct Effects of Two Dominant Enteric Bacteria on the Developmental Performance of *Spodoptera frugiperda* and Their Association with Physiological Metabolism. Sci. Rep..

[51] Kong H.G., Son J.-S., Chung J.-H., Lee S., Kim J.-S., Ryu C.-M. (2023). Population Dynamics of Intestinal *Enterococcus* Modulate *Galleria mellonella* Metamorphosis. Microbiol. Spectr..

[52] Zhu X., Li J., He A., Gurr G.M., You M., You S. (2024). Developmental Shifts in the Microbiome of a Cosmopolitan Pest: Unraveling the Role of *Wolbachia* and Dominant Bacteria. Insects.

[53] Zhang J., Gao S., Zheng F., Wang N. (2022). Intestinal Bacterial Diversity and Functional Analysis of Three Lepidopteran Corn Ear Worm Larvae. Insects.

[54] Wu L., Hu C., Liu T. (2024). Metagenomic Profiling of Gut Microbiota in Fall Armyworm (*Spodoptera frugiperda*) Larvae Fed on Different Host Plants. BMC Microbiol..

[55] Douglas A.E. (2013). Microbial Brokers of Insect-Plant Interactions Revisited. J. Chem. Ecol..

[56] Chen Y., Zhou H., Lai Y., Chen Q., Yu X.-Q., Wang X. (2021). Gut Microbiota Dysbiosis Influences Metabolic Homeostasis in *Spodoptera frugiperda*. Front. Microbiol..

