# Supplementary material for: Bacterial Communities Associated with Whole Eggs and Gut Tissues of Heortia vitessoides Moore (Lepidoptera: Crambidae) Across Developmental Stages and Sexes
Source: Insects. 2026 Jun 26;17(7):668. doi: 10.3390/insects17070668 (PMC13410662; doi:10.3390/insects17070668)
Supplement: Supplementary file 1 [file insects-17-00668-s001.zip › insects-4330307-supplementary.pdf]

Table S1. Alpha diversity summaries and statistical tests among sampled groups

A. Group-level summary of alpha-diversity indices (mean ± SD)

Index	Group	Mean ± SD	Minimum	Maximum
ACE	E	239.93 ± 82.06	167.00	328.79
ACE	L	165.09 ± 76.18	100.26	249.00
ACE	MP	43.26 ± 12.02	34.72	57.00
ACE	FP	103.67 ± 17.39	84.00	117.00
ACE	M	100.00 ± 13.45	89.00	115.00
ACE	F	22.00 ± 10.54	11.00	32.00
Chao	E	239.89 ± 82.00	167.00	328.67
Chao	L	165.00 ± 76.30	100.00	249.00
Chao	MP	42.86 ± 12.33	34.33	57.00
Chao	FP	103.67 ± 17.39	84.00	117.00
Chao	M	100.00 ± 13.45	89.00	115.00
Chao	F	22.00 ± 10.54	11.00	32.00
Shannon	E	3.899 ± 0.232	3.692	4.150
Shannon	L	3.242 ± 0.120	3.107	3.335
Shannon	MP	1.581 ± 0.232	1.348	1.813
Shannon	FP	2.664 ± 0.226	2.424	2.873
Shannon	M	1.884 ± 0.190	1.745	2.100
Shannon	F	0.754 ± 0.531	0.142	1.077
Simpson	E	0.043 ± 0.002	0.041	0.045
Simpson	L	0.096 ± 0.042	0.069	0.145
Simpson	MP	0.311 ± 0.009	0.301	0.319
Simpson	FP	0.133 ± 0.020	0.113	0.154
Simpson	M	0.313 ± 0.108	0.246	0.438
Simpson	F	0.596 ± 0.318	0.388	0.963
Sobs	E	239.67 ± 81.64	167.00	328.00
Sobs	L	165.00 ± 76.30	100.00	249.00
Sobs	MP	42.67 ± 12.50	34.00	57.00
Sobs	FP	103.67 ± 17.39	84.00	117.00
Sobs	M	100.00 ± 13.45	89.00	115.00
Sobs	F	22.00 ± 10.54	11.00	32.00

B. Global Kruskal–Wallis tests

Index	Test	Statistic	P value	Significance
ACE	Kruskal-Wallis	14.8713	0.0109	*
Chao	Kruskal-Wallis	14.8713	0.0109	*
Shannon	Kruskal-Wallis	16.2515	0.0062	**
Simpson	Kruskal-Wallis	15.502924	0.008416	**
Sobs	Kruskal-Wallis	14.871345	0.0109	*

Table S2. PERMANOVA and betadisper results based on Bray − Curtis dissimilarities

Analysis	Groups included	PERMANOVA R^2	PERMANOVA F	PERMANOVA P	betadisper F	betadisper P
All groups	E, L, MP, FP, M, F	0.5330	2.7380	0.0001	0.6370	0.6730
Gut-only	L, MP, FP, M, F	0.4960	2.4580	0.0016	0.7290	0.5880

Table S3. Candidate differentially abundant ASVs identified by ALDEx2 and their taxonomic annotations

ASV	Genus	kw.ep	kw.eBH	glm.ep	glm.eBH	Highest group	Highest mean relative abundance
ASV2	*Pseudomonas*	2.226E-02	7.780E-01	1.524E-05	1.132E-03	FP	0.1232
ASV138	*Acinetobacter*	1.174E-02	7.780E-01	4.759E-05	2.481E-03	L	0.0452
ASV9	*Agrobacterium*	2.029E-02	7.780E-01	2.896E-05	3.124E-03	L	0.0248
ASV4	*Methylobacterium*	1.167E-02	7.780E-01	6.831E-05	3.398E-03	FP	0.1491
ASV13	*Acinetobacter*	2.708E-02	7.780E-01	1.920E-04	7.519E-03	MP	0.0166
ASV180	*Corynebacterium*	2.070E-02	7.780E-01	3.716E-04	1.002E-02	E	0.0030
ASV1163	*Massilia*	7.725E-02	7.780E-01	5.785E-04	1.359E-02	E	0.0407
ASV22	*Pseudomonas*	2.619E-02	7.780E-01	4.939E-04	1.455E-02	L	0.0142
ASV183	*Brevundimonas*	3.102E-02	7.780E-01	8.630E-04	1.623E-02	E	0.0063
ASV56	*Methylobacterium*	8.882E-02	7.782E-01	7.959E-04	1.684E-02	E	0.0277
ASV53	*Brevundimonas*	3.880E-02	7.780E-01	7.718E-04	1.712E-02	E	0.0052
ASV36	*Herbaspirillum*	2.894E-02	7.780E-01	1.182E-03	1.918E-02	FP	0.0089
ASV27	*Sphingobium*	2.784E-02	7.780E-01	1.161E-03	1.995E-02	FP	0.0033
ASV175	*Acinetobacter*	2.612E-02	7.780E-01	1.456E-03	2.446E-02	L	0.0521
ASV994	*Methylobacterium*	8.719E-02	7.784E-01	1.598E-03	2.706E-02	E	0.0607
ASV5	*Methylobacterium*	2.225E-02	7.780E-01	1.170E-03	3.080E-02	FP	0.0964
ASV34	*Bradyrhizobium*	3.837E-02	7.780E-01	1.797E-03	3.438E-02	L	0.0046
ASV50	*Sphingomonas*	4.117E-02	7.780E-01	2.581E-03	3.708E-02	E	0.0027
ASV8	*Acinetobacter*	5.037E-02	7.780E-01	1.369E-03	3.752E-02	L	0.1269
ASV19	*Acinetobacter*	2.710E-02	7.780E-01	2.369E-03	3.923E-02	L	0.0489
ASV24	*Sphingobium*	3.241E-02	7.780E-01	4.054E-03	4.035E-02	L	0.0022
ASV38	*Acinetobacter*	2.050E-02	7.780E-01	2.850E-03	4.358E-02	L	0.0353
ASV1161	*unclassified Comamonadaceae*	8.892E-02	7.782E-01	4.508E-03	4.575E-02	E	0.0046
ASV189	*Stenotrophomonas*	3.000E-02	7.782E-01	3.892E-03	4.595E-02	L	0.0018
ASV35	*unclassified Comamonadaceae*	7.884E-02	7.780E-01	4.743E-03	4.759E-02	MP	0.0012
ASV179	*Brevundimonas*	3.890E-02	7.780E-01	4.823E-03	4.915E-02	L	0.0029

Table S4. NSTI values used to evaluate PICRUSt2 functional prediction

A. ASV-level NSTI distribution summary

Metric	Value	Percentage/Note
Number of ASVs	1209	
Mean NSTI	0.7137	
Standard deviation	4.6909	
Median NSTI	0.0950	
Q1	0.0355	
Q3	0.2109	
Interquartile range	0.035–0.211	Q1–Q3
Minimum	0.0001	
Maximum	82.0819	
ASVs with NSTI ≤ 0.10	621.0000	0.5136
ASVs with NSTI ≤ 0.20	876.0000	0.7246
ASVs with NSTI ≤ 0.30	1012.0000	0.8371
ASVs with NSTI ≤ 0.50	1118.0000	0.9247
ASVs with NSTI > 0.50	91.0000	0.0753

B. Group-level abundance-weighted NSTI summary

Group	Group description	Mean ± SD	Minimum	Maximum
E	Whole eggs	0.1134 ± 0.0448	0.0719	0.1608
L	Larval gut	0.0911 ± 0.0422	0.0577	0.1385
MP	Male pupal gut	0.0744 ± 0.0354	0.0361	0.1060
FP	Female pupal gut	0.0922 ± 0.0758	0.0430	0.1795
M	Male adult gut	0.0384 ± 0.0002	0.0382	0.0386
F	Female adult gut	0.0432 ± 0.0115	0.0327	0.0555
